# Supplementary material for: Site-specific identification and quantitation of endogenous SUMO modifications under native conditions
Source: Nat Commun. 2017 Oct 27;8:1171. doi: 10.1038/s41467-017-01271-3 (PMC5660086; doi:10.1038/s41467-017-01271-3)
Supplement: Supplementary file 1 — Supplementary Information [file 41467_2017_1271_MOESM1_ESM.docx]

**Supplementary Information Lumpkin et al**

**
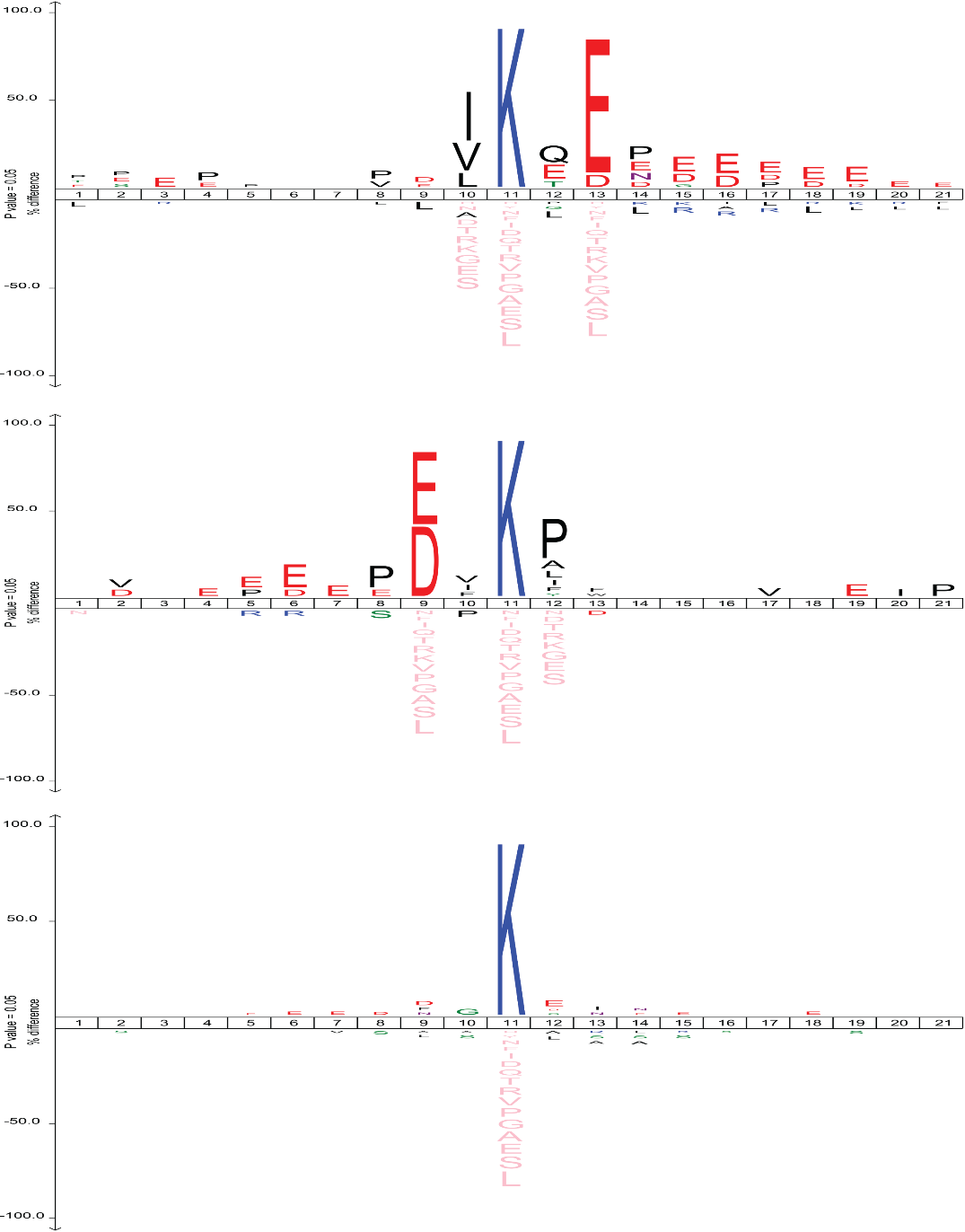
**

**Supplementary Figure 1**. “IceLogo” motif analysis of the KGG sites discovered using WaLP digestion from Hela and HCT116 cells. SUMO attachment at the “forward” sequence motif (ΨKX[E|D], where Ψ indicates a hydrophobic amino acid) (a) occurred in 31% of identified sites, at the “inverted” motif ([E|D]XK) (b) in 9% identified sites, and at other sites (c) in 60% of the identified sites.

**

**

**Supplementary Fig 2.** Gene ontology enrichment analysis of all proteins identified with SUMOylation sites from Hela and HCT116 cells. Depicted is the -log10 enrichment of selected enriched GO-biological processes (top) or GO-cellular compartments (bottom) using the predicted human proteome as background.
