# Supplementary material for: Site-specific identification and quantitation of endogenous SUMO modifications under native conditions
Source: Nat Commun. 2017 Oct 27;8:1171. doi: 10.1038/s41467-017-01271-3 (PMC5660086; doi:10.1038/s41467-017-01271-3)
Supplement: Supplementary file 3 — Descriptions of Additional Supplementary Files [file 41467_2017_1271_MOESM3_ESM.pdf]

Descriptions of Additional Supplementary Files

File Name: Supplementary Dataset 1
Description: Data table for all SUMO sites identified from mammalian cells

File Name: Supplementary Dataset 2
Description: Data table for all SUMO sites identified from tissue samples
